# Aortic flow and wall shear stress in aortic stenosis is associated with left ventricular remodeling

**DOI:** 10.1186/1532-429X-18-S1-Q57

**Published:** 2016-01-27

**Authors:** Florian von Knobelsdorff-Brenkenhoff, Achudhan Karunaharamoorthy, Ralf F Trauzeddel, Alex J Barker, Edyta Blaszczyk, Michael Markl, Jeanette Schulz-Menger

**Affiliations:** 1 Charité Medical Faculty and HELIOS clinics, Working group Cardiovascular MRI, Berlin, Germany; 2 Department of Biomedical Engineering, McCormick School of Engineering, Northwestern University, Chicago, IL USA; 3 Department of Radiology, Medical Physics, Feinberg School of Medicine, Northwestern University, Chicago, IL USA

## Background

Aortic stenosis (AS) can lead to highly variable stress for the left ventricle (LV) and consequently a broad range of LV remodeling. We hypothesize that changes in aortic blood flow caused by AS can contribute to cardiac afterload. The aim of this study was to describe the blood flow patterns in the ascending aorta in patients with AS and to determine its association with LV remodeling.

## Methods

Thirty-seven patients with AS (14 mild, 8 moderate, 15 severe, age 63 ± 13 years) and 37 healthy, age- and gender-matched controls (age 60 ± 10 years) were prospectively enrolled to undergo 4D-flow MRI of the aorta. Vorticity and helicity were graded as absent, mild or marked using time-resolved 3D pathlines. Eccentricity of the peak blood flow velocity was evaluated as absent, mild or marked using a 2D plane in the mid-ascending aorta, and flow displacement from the vessel center was calculated. Peak systolic wall shear stress (WSS_peak_) was quantified at the sinotubular level, mid-ascending and distal ascending aorta. LV remodeling was defined as increased ratio of LV mass by enddiastolic volume (relative wall mass, RWM).

## Results

**Figure 1:**
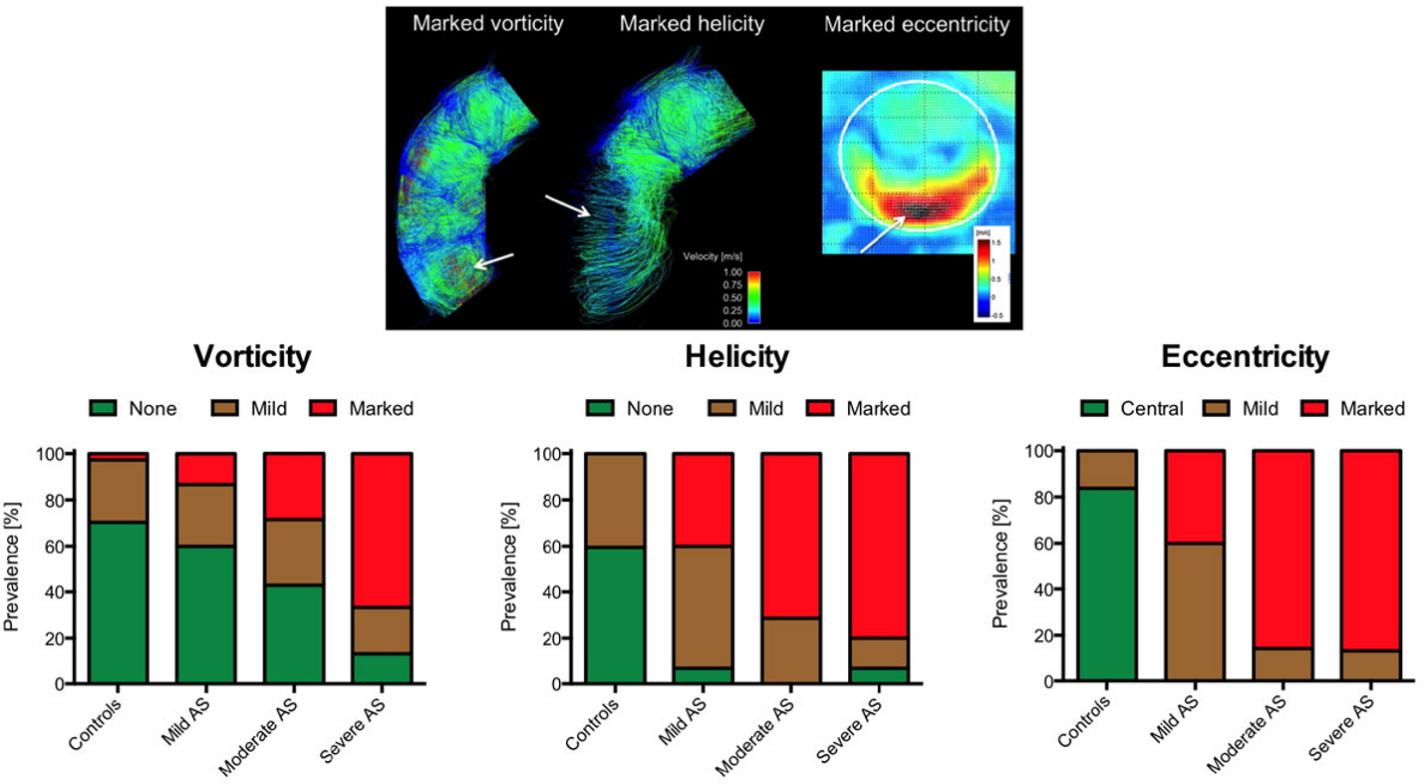
**Streamline examples (top) and qualitative flow grading (bottom) for helicity, vorticity and eccentricity**.

**Figure 2:**
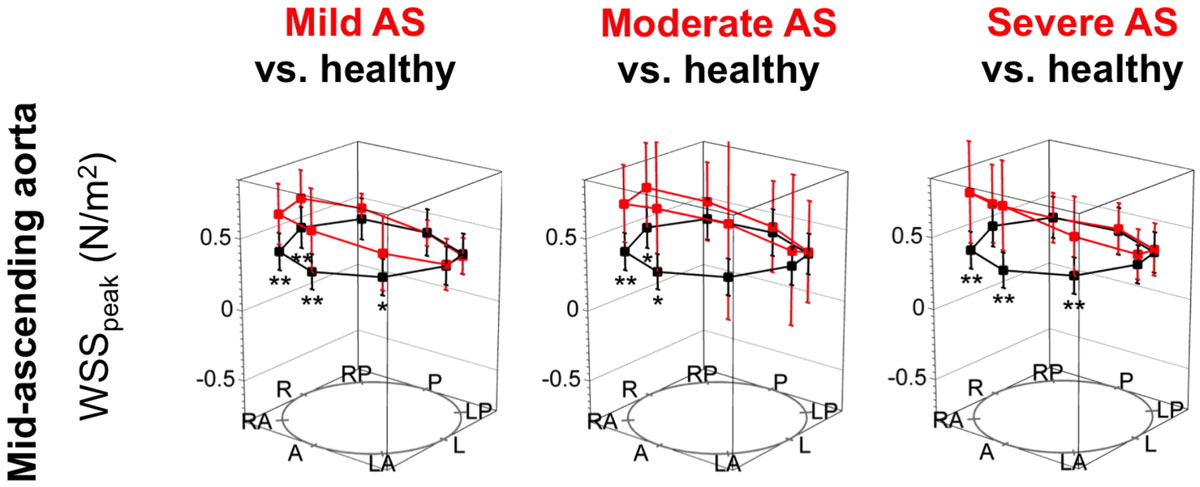
**WSS**_**peak**_ **distribution along the circumference of the mid-ascending aorta**. Comparison of the AS severity grades with healthy controls. A = anterior, LA = left anterior, L = left, LP = left posterior, P = posterior, RP = right posterior, R = right, RA = right anterior. "*" and "**" mark significant differences between the groups with p < 0.05 and p < 0.001.

The prevalence of helicity, vorticity and eccentricity was significantly higher in AS than in controls (Figure 1). In AS, aortic orifice area correlated significantly with the extent of eccentricity (r = -0.48; p = 0.003) and vorticity (r = -0.52; p = 0.001), and RWM correlated significantly with helicity, vorticity and eccentricity (r = 0.48, r = 0.60, r = 0.49; each p < 0.001). Patients with LV remodeling exhibited significantly higher normalized flow displacement than patients without LV remodeling (0.19 ± 0.05 vs. 0.15 ± 0.04; p = 0.034). WSS_peak_ was significantly elevated compared to controls in all severity grades of AS, with asymmetric peaks at the right and anterior curvature at the sinutubular level and in the mid-ascending aorta (Figure 2). There was no difference in WSS_peak_ levels between patients with and without LV remodeling.

## Conclusions

AS leads to significantly abnormal vorticity, helicity and eccentricity as well as WSS_peak_ distribution in the ascending aorta. LV remodeling correlated with the extent of abnormal blood flow.

